# Ultrasound Evaluation of Achilles Tendon Thickness in Diabetic Patients With and Without Foot Complications

**DOI:** 10.7759/cureus.62831

**Published:** 2024-06-21

**Authors:** Rishi Prajwal, Anil Kumar Sakalecha, Anees Dudekula, Nishanth Varma

**Affiliations:** 1 Radiodiagnosis, Sri Devaraj Urs Medical College, Kolar, IND

**Keywords:** diabetes mellitus type 2, achilles tendon thickness, achilles tendon (at), thickness, foot complications, ultrasound, diabetes mellitus

## Abstract

Introduction

Diabetes mellitus (DM) is a multifaceted metabolic disorder distinguished by elevated blood sugar levels. Type 2-DM (T2DM) stands as a significant contributor to disability due to its widespread occurrence of microvascular and macrovascular complications. According to certain researchers, prolonged elevated blood sugar levels have been observed to trigger a sequence of irregular alterations in the Achilles tendon (AT). AT thickness is one such indicator of these alterations.

Methods

This was a prospective study carried out on 51 individuals which was further divided into 3 groups (Group A - Normal individuals, Group B - DM without foot complications, Group C - DM with foot complications) with 17 individuals in each as healthy, DM with foot complications and without complications at Sri Devaraj Urs Medical College over a period of one year. The patients' demographics, basic medical records, and laboratory test results were examined and analyzed.

Results

The mean age of the study participants was 55.41 + 10.25 years. There was no significant difference in age and gender between the three groups. There was a significant difference in mean AT thickness between the groups (p<0.05). The mean thickness of AT was higher in Group C compared to Group B. Group A had the least AT thickness. However, there was no correlation between the variables in individual groups.

Conclusions

Modifications in the AT's structure may occur before the onset of foot and ankle issues in individuals with diabetes. Hence, the thickening of the AT can be used as an early indicator of impending diabetic foot complications.

## Introduction

The type 2 diabetes mellitus (T2DM) condition is a multifaceted and enduring metabolic disorder distinguished by elevated blood sugar levels. It ranks among the most prevalent chronic ailments encountered in clinical settings, with the potential to give rise to complications affecting multiple organs [1,2]. The process of nonenzymatic protein glycation is a significant contributor to the emergence of complications associated with DM [3]. In 2021, the International Diabetes Federation (IDF) reported around 537 million adults aged between 20 and 80 years as diagnosed with diabetes [4]. In India, according to National Family Health Survey-5 (NFHS-5) data, 13.5% of women and 15.6% of men are suffering from T2DM and are on medication [5].

As per the Centers for Disease Control and Prevention (CDC), approximately 95% of all diagnosed cases in adults are attributed to T2DM. T2DM stands as a significant contributor to disability because of its widespread occurrence of microvascular and macrovascular complications [6]. The incidence of rheumatologic disorders is higher in individuals with DM when compared to normal individuals [7-9]. According to certain researchers, prolonged elevated blood sugar levels have been observed to trigger a sequence of irregular alterations in the Achilles tendon (AT), ultimately resulting in tendinopathy with an increase in AT thickness as diabetes develops and progresses [10-13].

Growing evidence suggests that the lack of flexibility in the AT could play a role in the heightened forefoot pressure, potentially triggering the pathological cascade and leading to the development of foot ulcers [14-16]. MRI and ultrasound (US) are currently considered the most reliable methods for diagnosing AT disease [17,18]. US is effective for evaluating the mechanical properties of soft tissues, but its applicability to tendons has certain limitations.

The purpose of this study is to evaluate AT thickness in normal individuals and diabetic patients with complications and without foot complications and prove its role in early diagnosis of impending diabetic foot complications.

## Materials and methods

Study design, sample size, and source of data

This prospective comparative study was conducted on 51 individuals. The study consists of three groups with 17 individuals in each group as follows, Group A (normal individuals), Group B (DM patients without foot complications), and Group C (DM patients with foot complications). Healthy individuals who were matched in terms of age, gender, and body mass index (BMI) were chosen from the hospital staff to act as a control group (Group A). Patients with diabetes admitted to diabetes clinics between July 2022 and July 2023 were enrolled. The study was conducted in the Department of Radiodiagnosis at Sri Devaraj Urs Medical College.

Inclusion and exclusion criteria

All diabetic patients with and without foot complications were included in this study. Age- and gender-matched nondiabetic patients were selected as controls. Patients with a history of foot trauma, foot surgery, or Achilles tendinitis from other causes were excluded from the study. Athletes and patients who have other foot conditions were excluded from the study.

Method of data collection

The study obtained approval from the Institutional Ethical Committee of Sri Devaraj Urs Academy of Higher Education and Research with the approval number SDUMC/KLR/IEC/573/2023-24. Informed consent was obtained from all participants in the study.

Following the criteria mentioned earlier, our study included a total of 51 patients (17 in each group). We collected comprehensive clinical information from the patients, including their age, gender, anthropometric measures (e.g., height, weight, and BMI), duration of diabetes, and drugs that were being used were recorded. Pertinent medical history with symptoms such as numbness, tingling, and sensory loss in the feet indicative of peripheral neuropathy (PN), the presence of foot ulcers, comorbid conditions, and diabetes-related complications. Additionally, we documented their fasting blood sugar (FBS) and postprandial blood sugar (PPBS).

Patients and healthy individuals were subjected to sonographic examination using the Phillips EPIQ 5 (Philips Ultrasound Inc., Bothell, WA) machine with a high-frequency linear array transducer (L5-12 MHz). The examination involved evaluating both ATs in a prone position, with the patient's foot relaxed and hanging over the examination bed. To ensure good ultrasound contact, a larger quantity of coupling gel was applied, considering the AT's shallow location. The thickest portion of the AT was measured in the longitudinal plane.

Statistical analysis

The data collected were analyzed using the Statistical Product and Service Solutions (SPSS, version 20; IBM SPSS Statistics for Windows, Armonk, NY). The normality of the data was assessed by the Kolmogorov-Smirnov test. Socio-demographic variables were analyzed in terms of mean, standard deviation (SD), frequency (n), and percentage (%). A chi-square test was used for categorical variables. An ANOVA test was performed to compare the continuous variables of the three groups. A P value of <0.05 was taken as statistically significant. Data results were represented in the form of tables and figures.

## Results

Patient demographic characteristics

­The demographic characteristics of all three groups were compared, as shown in Table 1. There was no statistically significant difference in age and gender (p > 0.05).

**Table 1:** Patient demographic characteristics SD: Standard deviation; Group A: Healthy individuals; Group B: Diabetes mellitus without foot complications; Group C: Diabetes mellitus with foot complications

Variables	Group A	Group B	Group C	P value
Age (Mean + SD)	54.35 ± 13.6	55.88 ± 10.78	56 ± 10.25	0.877
Gender, n (%)				
Male	12 (70.6%)	12 (70.6%)	14 (82.4%)	0.662
Female	5 (29.4%)	5 (29.4%)	3 (17.6%)	

Comparison of laboratory tests

We conducted fasting blood sugar (FBS) and PPBS tests. There was a statistically significant difference in the mean FBS and PPBS levels between the three groups, as shown in Table 2.

**Table 2:** Comparison of laboratory tests *p < 0.05 (significant); FBS: Fasting blood sugar; PPBS: Postprandial blood sugar; SD: Standard deviation; Group A: Healthy individuals; Group B: Diabetes mellitus without foot complications; Group C: Diabetes mellitus with foot complications

Variables	Group A	Group B	Group C	P value
FBS	89.04 ±5.37	145.56 ± 14.95	161.13 ± 11.88	< 0.0001*
PPBS	124.06 ± 9.42	228.35 ± 21.25	236.88 ± 21.43	< 0.0001*

Comparison of mean thickness of AT

The mean thickness of the AT between the three groups was compared, as shown in Table 3. There was a statistically significant difference in the thickness of AT between the three groups at p < 0.001. The thickness of AT in group A is less compared to groups B and C.

**Table 3:** Comparison of mean thickness of Achilles tendon *p < 0.05 (significant); AT: Achilles tendon; SD: Standard deviation; Group A: Healthy individuals; Group B: Diabetes mellitus without foot complications; Group C: Diabetes mellitus with foot complications

Variables	Group A	Group B	Group C	P value
Thickness of AT (mm)	6.71 ± 0.77	8.71 ± 0.47	9.53 ± 0.51	< 0.0001*

**Figure 1:**
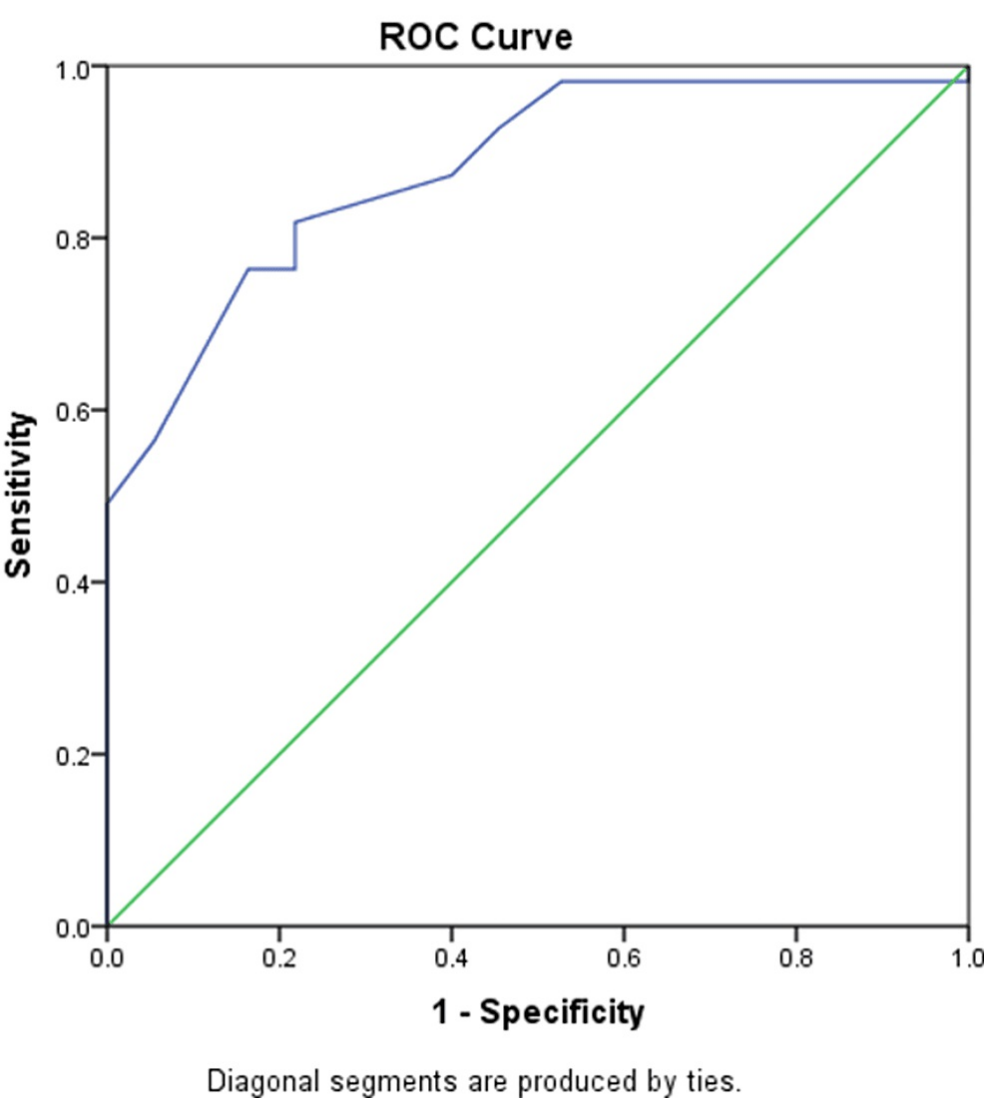
The receiver operator characteristic (ROC) curve showing the area under curve (AUC) for the Achilles tendon thickness as a marker for diabetic foot complications

ROC curves were generated to assess the sensitivity and specificity of AT's thickness as a marker of foot complications in diabetic patients (Figure 1). The optimal results of the cutoff value of the thickness of 8.95 mm are highly accurate with excellent sensitivity and specificity. At this threshold, the specificity was 78.18% and the sensitivity was found to be 81.8%.

**Figure 2:**
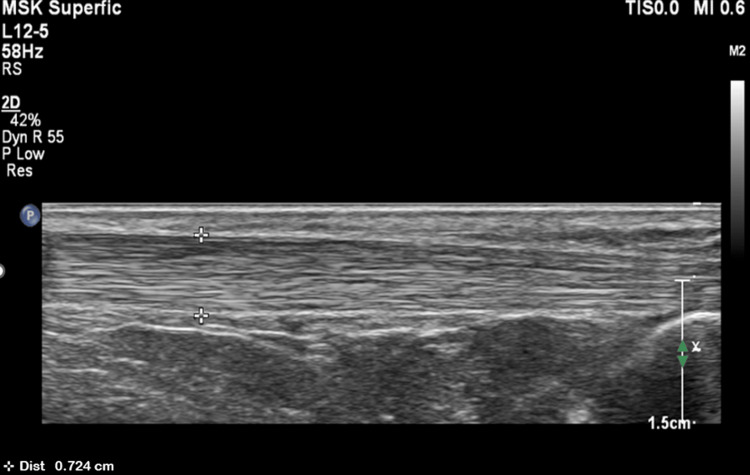
A greyscale ultrasound image showing a normal Achilles tendon using a linear probe (5-12 MHz)

Figure 2 shows a greyscale ultrasound image of a normal AT.

## Discussion

The AT is the human body's largest, thickest, and most robust tendon. Its crucial role in foot biomechanics, along with the plantar fascia, is indispensable. Prior research has indicated that individuals with DM exhibit an increase in the AT's thickness [9]. These alterations are more pronounced in individuals who have neuropathic complications and a history of prior foot ulcers, but they can also be observed in individuals without any diabetic-related complications. Consequently, identifying Achilles tendinopathy at an early stage in individuals with T2DM may have a role in averting the development of diabetic foot ulcers.

In the current study, we included 51 participants with 17 in each group. The mean age of overall participants was 55.41 ± 10.25 years with a 1-2 standard deviation in each group. In group A, the male-to-female ratio was 12:5 in group A, 12:5 in group B, and 14:3 in group C. Similarly, in a study conducted by Evranos et al., which included three groups (the control group, DM with ulcer group, and DM without ulcer groups); the overall mean age and individual groups were similar to our study, but the gender ratio was low compared to our study [10].

In this study, we concluded significant thickening of the AT in patients having T2DM with foot ulcers and without foot ulcers compared to normal healthy individuals with a mean thickness of 8.71 ± 0.47mm in T2DM without foot ulcers and 9.53 ± 0.51 mm in T2DM with foot ulcers. Moreover, we found significant thickening of AT in diabetic patients with foot ulcers compared to diabetic patients without foot ulcers.

The increase in the AT's thickness reached statistical significance among patients with neuropathy and those with a history of neuropathic ulcers when compared to the control group. Cheing et al. demonstrated that individuals with diabetes exhibited a thicker AT compared to the healthy control subjects [19].

Our study yielded results that align with the findings of Giacomozzi et al. [20]. However, it is important to note a distinction: our patients had ongoing foot ulcers, whereas the participants in Giacomozzi et al.'s study had a history of previous neuropathic ulcers [20]. Given the resemblance between our study group, which had ulcers, and the group of diabetes patients with prior neuropathic ulcers studied by Giacomozzi et al., the consistency in results appears to be more than coincidental.

A hypothesis has been proposed suggesting that nonenzymatic glycation of the collagenous component because of hyperglycemia plays a role in this thickening. Additionally, the increased mechanical load on the foot, often observed in individuals with diabetes because of higher body mass, may contribute to this phenomenon [21]. D'Ambrogi et al. proposed that the AT's thickness exhibited a significant increase in individuals with neuropathy [22].

The findings from this study substantiate the hypothesis that DM induces alterations in the AT. We took diligent measures to mitigate potential issues by adhering to established protocols, including thorough training, before initiating the study.

The study had a few limitations. The sample size was small, as the study was confined to a single center, and the findings cannot be generalized to a larger community. We do not have follow-up data from the subjects that could have influenced the results. Further longitudinal multicentric studies with large samples are recommended to support this study’s findings.

## Conclusions

In conclusion, our findings indicate that modifications in the AT structure may occur before the onset of foot and ankle issues in individuals with diabetes. AT is significantly thickened in diabetic patients with foot ulcers compared to diabetic patients without foot ulcers. Hence, the thickening of the AT can be used as an early indicator of impending diabetic foot complications. Early detection of impending foot complications will help in reducing associated morbidity and hence help improve quality of life.

## References

[1] American Diabetes Association (2020). 11: microvascular complications and foot care: standards of medical care in diabetes-2020. Diabetes Care.

[2] Qiu H, Yang H, Yang Z, Yao Q, Duan S, Qin J, Zhu J (2022). The value of radiomics to predict abnormal bone mass in type 2 diabetes mellitus patients based on CT imaging for paravertebral muscles. Front Endocrinol (Lausanne).

[3] Mentink CJ, Hendriks M, Levels AA, Wolffenbuttel BH (2002). Glucose-mediated cross-linking of collagen in rat tendon and skin. Clin Chim Acta.

[4] (2024). IDF diabetes atlas 10th edition. https://diabetesatlas.org/data/en/.

[5] NHFS NHFS (2024). National Family Health Survey, India. MOHFW.

[6] Frykberg RG (1998). Diabetic foot ulcers: current concepts. J Foot Ankle Surg.

[7] Abate M, Schiavone C, Pelotti P, Salini V (2010). Limited joint mobility in diabetes and ageing: recent advances in pathogenesis and therapy. Int J Immunopathol Pharmacol.

[8] Abate M, Schiavone C, Salini V (2010). Sonographic evaluation of the shoulder in asymptomatic elderly subjects with diabetes. BMC Musculoskelet Disord.

[9] de Oliveira RR, Lemos A, de Castro Silveira PV, da Silva RJ, de Moraes SR (2011). Alterations of tendons in patients with diabetes mellitus: a systematic review. Diabet Med.

[10] Evranos B, Idilman I, Ipek A, Polat SB, Cakir B, Ersoy R (2015). Real-time sonoelastography and ultrasound evaluation of the Achilles tendon in patients with diabetes with or without foot ulcers: a cross sectional study. J Diabetes Complications.

[11] Akturk M, Karaahmetoglu S, Kacar M, Muftuoglu O (2002). Thickness of the supraspinatus and biceps tendons in diabetic patients. Diabetes Care.

[12] Batista F, Nery C, Pinzur M (2008). Achilles tendinopathy in diabetes mellitus. Foot Ankle Int.

[13] Akturk M, Ozdemir A, Maral I, Yetkin I, Arslan M (2007). Evaluation of Achilles tendon thickening in type 2 diabetes mellitus. Exp Clin Endocrinol Diabetes.

[14] Rao SR, Saltzman CL, Wilken J, Yak HJ (2006). Increased passive ankle stiffness and reduced dorsiflexion range of motion in individuals with diabetes mellitus. Foot Ankle Int.

[15] Salsich GB, Mueller MJ, Hastings MK, Sinacore DR, Strube MJ, Johnson JE (2005). Effect of Achilles tendon lengthening on ankle muscle performance in people with diabetes mellitus and a neuropathic plantar ulcer. Phys Ther.

[16] Trevino SG, Buford WL Jr, Nakamura T, John Wright A, Patterson RM (2004). Use of a torque-range-of-motion device for objective differentiation of diabetic from normal feet in adults. Foot Ankle Int.

[17] Aubry S, Nueffer JP, Tanter M, Becce F, Vidal C, Michel F (2015). Viscoelasticity in Achilles tendonopathy: quantitative assessment by using real-time shear-wave elastography. Radiology.

[18] Nallamshetty L, Nazarian LN, Schweitzer ME (2005). Evaluation of posterior tibial pathology: comparison of sonography and MR imaging. Skeletal Radiol.

[19] Cheing GL, Chau RM, Kwan RL, Choi CH, Zheng YP (2013). Do the biomechanical properties of the ankle-foot complex influence postural control for people with Type 2 diabetes?. Clin Biomech (Bristol, Avon).

[20] Giacomozzi C, D'Ambrogi E, Uccioli L, Macellari V (2005). Does the thickening of Achilles tendon and plantar fascia contribute to the alteration of diabetic foot loading?. Clin Biomech (Bristol, Avon).

[21] Duffin AC, Lam A, Kidd R, Chan AK, Donaghue KC (2002). Ultrasonography of plantar soft tissues thickness in young people with diabetes. Diabet Med.

[22] D'Ambrogi E, Giacomozzi C, Macellari V, Uccioli L (2005). Abnormal foot function in diabetic patients: the altered onset of Windlass mechanism. Diabet Med.

